# High Pressure Properties of a Ba-Cu-Zn-P Clathrate-I

**DOI:** 10.3390/ma9080692

**Published:** 2016-08-12

**Authors:** Juli-Anna Dolyniuk, Kirill Kovnir

**Affiliations:** Department of Chemistry, University of California, Davis, One Shields Avenue, Davis, CA 95616, USA; jdolyniuk@ucdavis.edu

**Keywords:** clathrate, high pressure, powder X-ray diffraction, bulk modulus

## Abstract

The high pressure properties of the novel tetrel-free clathrate, Ba_8_Cu_13.1_Zn_3.3_P_29.6_, were investigated using synchrotron powder X-ray diffraction. The pressure was applied using a diamond anvil cell. No structural transitions or decomposition were detected in the studied pressure range of 0.1–7 GPa. The calculated bulk modulus for Ba_8_Cu_13.1_Zn_3.3_P_29.6_ using a third-order Birch-Murnaghan equation of state is 65(6) GPa at 300 K. This bulk modulus is comparable to the bulk moduli of Ge- and Sn-based clathrates, like A_8_Ga_16_Ge_30_ (A = Sr, Ba) and Sn_19.3_Cu_4.7_P_22_I_8_, but lower than those for the transition metal-containing silicon-based clathrates, Ba_8_*T_x_*Si_46−*x*_, *T* = Ni, Cu; 3 ≤ *x* ≤ 5.

## 1. Introduction

Clathrates are unique crystalline compounds with crystal structures composed of covalently-bound 3D frameworks with large polyhedral cages encapsulating guest atoms. Thus, they are promising materials for thermoelectric applications since clathrates allow for the partial decoupling of heat and charge transport. Likewise, clathrates can be made from a wide range of elemental systems with guests ranging from Li to Tl, and framework components ranging from Li to Bi [1,2,3,4,5]. This elemental diversity generates a variety of properties, ensures enhanced structural tunability, and has furthered efforts to study clathrates for other scientific and engineering purposes [1,2,6]. In many cases, an applied pressure has also been shown to improve the properties of materials, sometimes drastically [7]. An example case is that of SmTe where, under applying a 4 GPa pressure, the thermoelectric performance was improved by five orders of magnitude due to an over 10^6^-fold decrease in its electrical resistivity [7,8].

One measure of the strength of a clathrate framework is its bulk modulus at different pressures, *K*, i.e., a material’s change in volume with pressure. Most clathrates exhibit room temperature bulk moduli in the range of 50–120 GPa, depending on their elemental components and their overall structures. Silicon-based clathrates, for example, tend to have higher bulk moduli and lower compressibilities than isostructural germanides [2].

Alternatively, few clathrates based on transition metal-phosphorus frameworks are known [3,4,5], and, to the best of our knowledge, no mechanical properties were reported for such systems. Our study of the quaternary clathrate-I Ba-Cu-Zn-P system showed that unlike the ternary clathrate Ba_8_Cu_16_P_30_ which crystallizes in the orthorhombic *Pbcn* space group due to superstructural ordering, the quaternary Ba_8_Cu_13.1_Zn_3.3_P_29.6_ clathrate crystallizes in a conventional clathrate-I cubic space group *Pm*-3*n* [9]. In this work we report a variable-pressure synchrotron X-ray powder diffraction investigation of Ba_8_Cu_13.1_Zn_3.3_P_29.6_.

## 2. Results and Discussion

### 2.1. Crystal Structure

Ba_8_Cu_13.1_Zn_3.3_P_29.6_ crystallizes in a cubic *Pm*-3*n* space group, which is typical for clathrate-I. Two different cage types are present in this crystal structure: smaller pentagonal dodecahedra and larger tetrakaidecahedra. The first is a smaller, 20-vertex cage made up of twelve five-membered faces, and the second is a larger, 24-vertex cage composed of twelve five- and two six-membered faces. In the case of Ba_8_Cu_13.1_Zn_3.3_P_29.6_, Ba atoms are encapsulated in cages composed of M and P atoms, M = Cu and Zn. All framework atoms in the clathrate-I framework are tetrahedrally coordinated.

**Figure 1:**
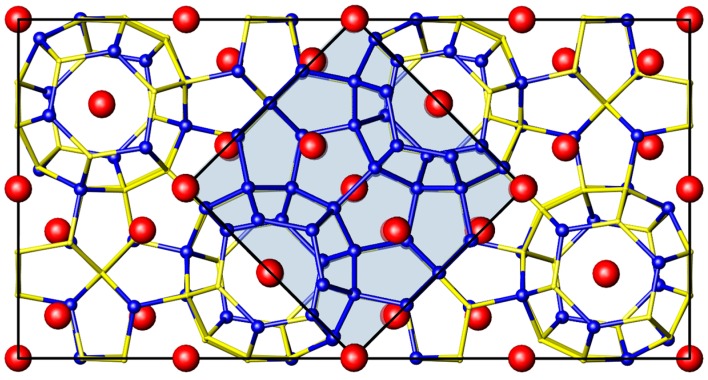
Clathrate-I unit cell of the large orthorhombic supercell with isolated M and P sites (blue, and yellow, respectively) and small cubic subcell (central blue square). Ba atoms are shown in red.

**Figure 2:**
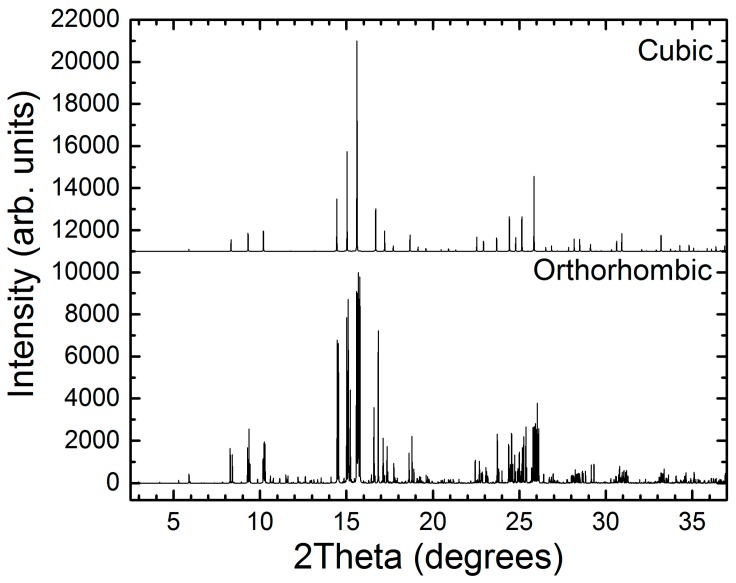
Calculated powder diffraction patterns (*λ* = 0.72775 Å) for cubic and orthorhombic structures of clathrate-I with identical composition Ba_8_Cu_16_P_30_.

In the conventional cubic structure of clathrate-I, there are two Ba sites and three framework sites of varying multiplicities: 6*c*, 16*i*, 24*k*. In the case of Ba_8_Cu_13.1_Zn_3.3_P_29.6_ these sites are jointly occupied by Cu, Zn, and P atoms (Figure 1). The superstructural ordering observed for the Zn-free compound, Ba_8_Cu_16_P_30_, results in a fourfold increase of the unit cell volume and a reduction of the symmetry to orthorhombic *Pbcn* due to a segregation of Cu and P atoms over different crystallographic sites, generating eight Cu sites and fifteen P sites [3]. In turn, this leads to a splitting of the powder diffraction peaks along with the appearance of new intermediate peaks. Thus, such a phase transformation is clearly detectable via powder X-ray diffraction (Figure 2).

### 2.2. Synchrotron X-ray Powder Diffraction

Powder patterns were collected at a wavelength of 0.72775 Å using a diamond anvil cell setup and internal standard, NaCl, for pressure calibration. At certain pressures, an overlap of the main peak of NaCl with one of the two most intense clathrate peaks was observed. These overlaps made Rietveld refinements challenging. Such refinements were performed by masking the overlapping portion of powder patterns, fitting the rest of the pattern, and unmasking the peaks and finalizing the refinement.

**Figure 3:**
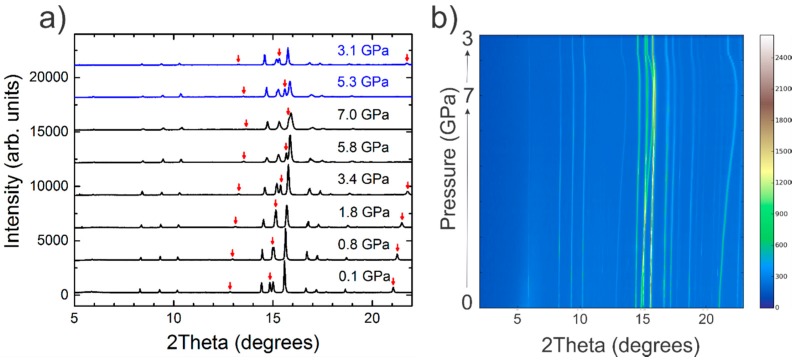
Selected room temperature synchrotron X-ray powder diffraction patterns are shown for various pressures (**a**) upon compression (black) and decompression (blue). The main peaks of the NaCl standard are indicated by red arrows; a contour plot of the collected diffraction patterns is shown in (**b**). White is the highest peak intensity and blue is the lowest.

Ba_8_Cu_13.1_Zn_3.3_P_29.6_ showed no structural transition and did not completely decompose over the whole range of applied pressures, 0.1–7 GPa (Figure 3). As the pressure was increased, the peaks softened and broadened, but no structural collapse was observed. Although, at the highest applied pressure, significant peak broadening was observed, potentially masking the orthorhombic peak splitting. After compression, the peak intensities decreased, and full peak intensities were not recovered upon decompression, probably due to the introduction of strains and defects or due to the partial amorphization of the main phase. The lack of a phase change and the absence of complete decomposition of Ba_8_Cu_13.1_Zn_3.3_P_29.6_ is in good agreement with the high-pressure studies of conventional Si- and Ge-based clathrates which do not undergo phase transitions until much higher pressures [10,11,12]. For example, the Ba_8_Si_39_Ge_7_ clathrate-I is stable until 14 GPa, and the clathrate-IX Ba_24_Si_100_ undergoes a phase transition at approximately 23 GPa [6,10]. Variable-pressure Raman studies, however, have indicated the possibilities of lower pressure transitions for other clathrate systems, which have been attributed to guest atoms’ rattling. In Ba_8_Si_39_Ge_7_, anharmonic rattling is proven by EXAFS which shows incoherent Ba displacements up to the pressure of the phase transition [6].

**Figure 4:**
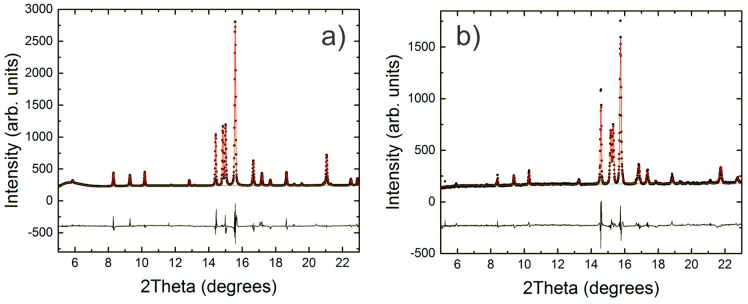
Rietveld refinements are shown for 0.1 GPa (**a**) and partially decompressed 3.1 GPa (**b**). The data are shown in black and the fit is shown in red. A difference curve is shown along the bottom.

A Rietveld refinement of the powder patterns was used to determine the unit cell parameters of the Ba_8_Cu_13.1_Zn_3.3_P_29.6_ clathrate at different pressures. Two examples of such pattern fittings are shown in Figure 4 for data collected at an initial compression of 0.1 GPa and for the post-compression sample, decompressed to 3.1 GPa. Changes in the relative intensities of the diffraction peaks were observed. The three most intense clathrate peaks in order of increasing intensity are *hkl* = 220, 320, 321 at 14.4, 15.0, and 15.6 degrees 2θ at 0.1 GPa, respectively. The observed change in the relative intensities of the 220 and 321 peaks may be attributed to possible redistributions of the Cu, Zn, and P atoms over three framework positions. Unfortunately, the quality and resolution of the powder patterns were not sufficient to determine the peculiarities of the crystal structure, and neutron or resonant X-ray diffraction would be necessary to distinguish Cu from Zn.

### 2.3. Bulk Modulus Calculations

**Figure 5:**
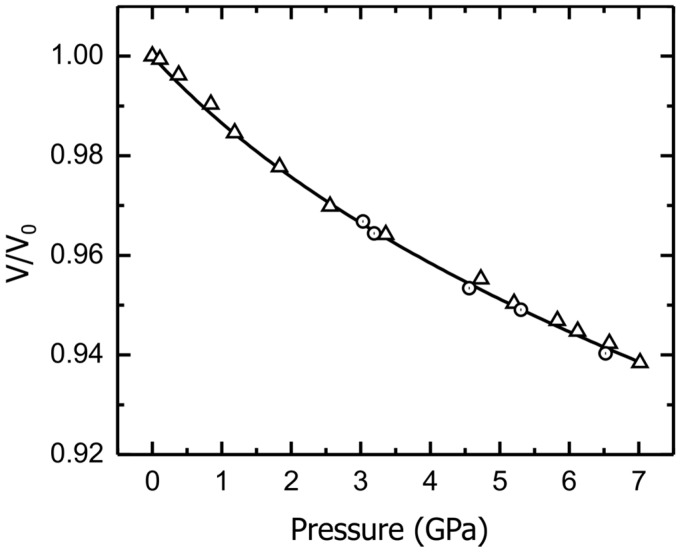
A Birch-Murnaghan fit is shown for the pressure dependence of the experimentally-obtained relative volumes of Ba_8_Cu_13.1_Zn_3.3_P_29.6_. Compression data: triangles; decompression data: circles.

A third-order Birch-Murnaghan equation of state was used to fit the pressure/volume data and determine *K*_0_ using EOSFit7-GUI [13,14,15,16]. The calculated *K*_0_ and *K*_p_ were 65(6) GPa and 19(3), respectively, at 300 K. Figure 5 shows the pressure dependence of the experimentally-obtained volume changes for Ba_8_Cu_13.1_Zn_3.3_P_29.6_. The whole dataset was fit using one curve. The decompression data fit the same curve as the compression data, and no obvious phase changes could be detected.

For the transition metal-doped tetrel clathrates, a general trend is that the bulk modulus will decrease with an increase in the transition metal content. For example, resonant ultrasound spectroscopy was used to predict the bulk moduli for Ba_8_Ni_3_Si_43_, Ba_8_Ni_3.3_Si_42.7_, and Ba_8_Cu_5_Si_41_ as 81.6, 83.3, and 75.5 GPa, respectively [2]. A similar trend was observed for Ge-based clathrates, i.e., Ba_8_Cu_5_Ge_41_, Ba_8_Zn_7_Ge_39_, Ba_8_Zn_8_Ge_38_, where the bulk moduli decreased from 64.0 to 56.1 to 55.6 GPa, respectively [2]. Such trends include compounds with different transition metals and need to be considered with care since, for compounds with the same transition metal such as Ni or Zn in the aforementioned examples, changes in the bulk modulus values are small. Lower bulk moduli for the Ge-based clathrates compared to the Si-based ones, Ba_8_Cu_5_Si_41_ (75.5 GPa) and Ba_8_Cu_5_Ge_41_ (64.0 GPa), can be correlated to the strength of framework bonds, as Si-Si bonds are stronger than Ge-Ge bonds [17,18].

To the best of our knowledge, no bulk moduli of the tetrel-free clathrates were reported. The large number of Zn-P, Cu-P, and P-P bonds in the structure of Ba_8_Cu_13.1_Zn_3.3_P_29.6_ differentiates this clathrate from conventional Si- or Ge-based clathrates, where tetrel-tetrel Si-Si or Ge-Ge bonds dominate in the framework. With a calculated bulk modulus of 65(6) GPa, Ba_8_Cu_13.1_Zn_3.3_P_29.6_ resists compression similarly to the other clathrate-I systems: Rb-deficient Rb_6.15_Si_46_ (*K*_0_ = 59 GPa), Sr_8_Ga_16_Ge_30_ (*K*_0_ = 64 GPa), and Ba_8_Ga_16_Ge_30_ (*K*_0_ = 67 GPa). One cationic clathrate, Sn_19.3_Cu_4.7_P_22_I_8_, where guests are iodine anions and the positively-charged framework is composed of Sn, Cu, and P atoms was predicted to exhibit a bulk modulus of 60.9 GPa [2].

## 3. Experimental Section

### 3.1. Synthesis

All manipulations of the starting materials were performed inside an argon-filled glove box (*p*(O_2_) < 1 ppm). The starting materials, metallic barium (Sigma-Aldrich, 99.9%, St. Louis, MO, USA), copper powder (Alfa Aesar, 99.99%, Ward Hill, MA, USA), zinc shavings (Alfa Aesar, 99.8%), and red phosphorus (Alfa Aesar, 99%) were used as received. Ba_8_Cu_13.1_Zn_3.3_P_29.6_ was synthesized by annealing the elements in a stoichiometric ratio in a carbonized silica tube sealed under vacuum. The sample was ramped to 1123 K over 17 h, held there for one week, and allowed to cool. The sample was then ground in an argon-filled glovebox and reannealed twice with a similar heating profile. No impurity phases were observed in the sample after its final annealing, and its composition was confirmed with scanning electron microscopy-energy dispersive X-ray spectroscopy (SEM-EDS).

### 3.2. High Pressure Synchrotron X-ray Powder Diffraction Collection and Analysis

High-pressure X-ray diffraction data were collected at beamline 17-BM at the Advanced Photon Source (APS) at Argonne National Lab (ANL). To prepare the sample, powder of the internal standard, NaCl, was mixed with a powder sample of the clathrate, and the mixed powders were then loaded into a diamond anvil cell (DAC) with Alfa Aesar silicone oil as a pressure-transmitting fluid.

Rietveld refinements of the variable-pressure data were performed using GSAS and EXPGUI [19,20,21,22]. The refined unit cell dimensions of NaCl were used to determine the pressure acting on the clathrate sample. Furthermore, EOSfit7-GUI was used to fit the experimental pressure/volume data of the clathrate to a third-order Birch-Murnaghan equation of state model and determine the bulk modulus of the clathrate at different pressures. In this model, the following formulas are used to calculate the bulk modulus, *K*, where *f_E_* is the finite Eulerian strain: $K_{PT}=K_{0T}{\left(1+2f_{E}\right)}^{5/2}\left(1+\left(3K_{0T}^{'}-5\right)f_{E}+\frac{27}{2}\left(K_{0T}^{'}-4\right)f_{E}^{2}\right)$ [13,14,15,16,23].

## 4. Conclusions

The tetrel-free clathrate, Ba_8_Cu_13.1_Zn_3.3_P_29.6_, exhibits high pressure stability comparable to the stabilities of Si- and Ge-based clathrates. No obvious decomposition or structural transitions were observed over the studied pressure range of 0.1–7 GPa, though, at the highest applied pressure, substantial line broadening may mask some structural distortions. As a result of the fitting of the relative volume-pressure dependence, the bulk modulus for Ba_8_Cu_13.1_Zn_3.3_P_29.6_ was calculated to be 65(6) GPa at 300 K. This bulk modulus is comparable to the bulk moduli of Ge- and Sn-based clathrates, which are in the range of 56–67 GPa. Clathrates based on Si-Ni or Si-Cu frameworks exhibit higher bulk moduli in the range of 75–83 GPa.
